# Fhit induces the reciprocal suppressions between Lin28/Let-7 and miR-17/92miR

**DOI:** 10.7150/ijms.51429

**Published:** 2021-01-01

**Authors:** Hae-Jung Chae, Jong Bae Seo, Sung-Hak Kim, Young-Jun Jeon, Sung-Suk Suh

**Affiliations:** 1 Department of Biosciences, Mokpo National University, Joennam 58554, South Korea; 2 Department of Biomedicine, Health & Life Convergence Science, BK21 Four, Mokpo National University, Joennam 58554, South Korea; 3 Lab of Animal Molecular Biochemistry, Chonnam National University, Gwangju, 61186, Republic of Korea.; 4 Department of Integrative Biotechnology, Sungkyunkwan University, Suwon 16419, South Korea.

**Keywords:** Fhit, miRNAs, tumor suppressor, Let-7, Lin28b

## Abstract

**Objective:** Fhit gene is known as a genome “caretaker” and frequently inactivated by deletion or hypermethylation on the promoter in several cancers. In spite of several lines of evidence, the exact mechanism underlying Fhit-induced biology is relatively less studied. This study will focus the role of Fhit in regulating Lin28 and microRNAs (miRNAs) loop.

**Material and Methods**: To this end, we employed Fhit overexpressing isogenic cell lines to conduct miRNA nanostring array, and differentially expressed miRNAs were identified. Using real-time PCR and Western blot analysis, expression levels of Lin28b or miRNAs were investigated in response to the overexpression of Fhit gene in H1299 lung cancer cells.

**Results:** A series of *in vitro* including gene nanostring analyses revealed that Lin28B protein was induced by Fhit gene overexpression, which consequently suppressed Let-7 miRNAs. Also, we found that miRNAs in miR-17/92 clusters are redundantly increased and there is an inverse correlation between Let-7 and miR-17/92 clusters in Fhit-expressing cells. Also, a series of in vitro experiments suggests that ELF-1- and/or STAT1-dependent Lin28b regulation is responsible for Let-7 induction in Fhit-expressing cancer cells.

**Conclusions:** Based on the same experimental system proving that Fhit gene has a robust role in suppressing tumor progression and epithelial-mesenchymal transition, our data show that Fhit mediates the negative feedback between Lin28/Let-7 axis and miR-17/-92 miRNA although the physiological relevance of current interesting observation should be further investigated.

## Introduction

Loss of heterozygosity (LOH) is a hall maker for a cancer, often found in multiple tumor suppressors and consequently enhances tumor progression. Fragile histidine triad (FHIT) gene straddling FRA3B at chromosome 3p14.2 region was first identified homozygously deleted gene in multiple cancer cell lines, which could be used for a diagnostic and/or prognostic marker for multiple cancers 1-3. Subsequent multiple evidences have suggested that FHIT plays an important role in suppressing cancer progression by regulating cell cycle, cell death, metastatic potential including epithelial-mesenchymal transition (EMT) as well as correlating global genomic instability with LOH of FHIT gene 4-7 Compared to the robust role in FHIT gene as a tumor suppressor, the mechanism underlying FHIT-induced biology is relatively less understood because of lack of information about the binding partner of FHIT protein as either tumor suppressor or oncogenic protein. The physiological role of FHIT is known as a typical dinucleoside 5',5"'-P1, P3-triphosphate (Ap3A) hydrolase, which seems to be associated with subcellular mRNA decay process 8, 9. Moreover, Kiss et al applied inducible Fhit gene expression system in a bona fide Fhit null cell line, H1299, and performed RNA-Seq and Ribo-Seq analysis to identify differential expression and/or ribosome occupancy in response to Fhit protein. Associated with Fhit expression impacted the genes responsible for lung cancer progression, consistent with the nucleoside triphosphatase functions function of Fhit protein to induce genomic instability in cancer context 10. However exact mechanism underlying Fhit overexpression in cancer progression is not well understood.

miRNA is classified as a small non coding RNA whose matured forms are generally 18-22 nt in length. The dysregulation of miRNA expression is a hall marker for a cancer, which consequently regulates cancer progression by suppressing their corresponding target genes 11. In spite of context dependency of most miRNAs found in different cancer types, let-7 family exhibited robust roles in the suppression of multiple solid tumor progression. The let-7 miRNA was first identified in C. *elegans* as an essential developmental gene, and the suppression of let-7 family has been reported in many cancer types 12, 13. The Lin-28 has two different paralogs known as an RNA binding protein to inhibit posttranscriptional maturation process of the let-7 miRNAs, which suppresses the tumor the roles of let-7 miRNA as a tumor suppressor. Also, let-7 miRNA suppresses Lin-28 expression through binding to its 3'UTR region, suggesting that there is double negative feedback between let-7 miRNA and Lin-28 proteins, which plays an important role in tumor suppression by targeting multiple oncogenic proteins such as Myc and Ras 14. miR-17/92 clusters encoded in C13orf25 gene and 6 different miRNAs including miR-17, -18, -19a, -19b, -20a and -92a differentially processed from 800 nts of mature C13orf25 transcripts 15. The miR-17/92 cluster was fist defined polycistronic onco-miRs and was found to be increased in expression and to accelerate myc-driven mice lymphomagenesis. Subsequent reports have convinced that miR-17/92 cluster was often dysregulated in several cancers, and induced tumor progression by targeting several tumor suppressors 15-17. Thus, miR-17/92 clusters have an oncogenic role, but let-7 family is a tumor suppressor, which seems to be functionally mutually exclusive in lung cancer 18. However, no mechanistic validation has been addressed yet.

Based on literature review, Fhit and let-7 family generally play a suppressive role in lung and colorectal cancer progression whereas Lin28 and miR-17/92 clusters enhances cancer progression 12-17. Although there were several reports suggesting that Let-7 and miR-17/-92 clusters play a critical role in cancer development in an opposite way, no study has suggested their direct relation. Moreover, no expressional and functional relationships have not been characterized in Lin28/Let-7 axis and miR-17/-92 clusters.

Here we first report that there is a cross talk between miR-17/92 clusters and let-7 family, mediated by Lin28 protein in lung and colorectal cancer. To our surprise, the negative regulation of let-7 family was paradoxically induced by Fhit gene overexpression.

## Materials and Methods

### Cell lines and Nucleic acid delivery

A549, HEK293 and HCT116 cells were originally purchased from the American Type Culture Collection (ATCC) right before starting this project. The cells were maintained in DMEM (Dulbecco Modified Eagle Medium) supplemented with 10% fetal bovine serum (FBS). The mycoplasma contamination was regularly monitored using PlasmoTest (Invivogen). Lipoectamine 3000 reagent for plasmid or Lipofectamine RNAiMax for siRNAs or miRNAs was used for transfection experiment.

### Lentivirus production and stable cell generation

Fhit cDNA was subcloned into pCDH lentiviral vector. The lentiviral constructs were co-transfected with packing constructs in HCT116 cells for 48 hrs. Subsequently the supernatant was collected and enriched by PEG-iTtm for 24 hours at 4 C. The viruses were collected by centrifugation of the samples at 1500G for 30mins at 4 C, followed by resuspending it using DMEM with 10% FBS.

### Antibodies for Western blot analysis and probes for qRT-PCR analysis

Samples were extracted in 15 mM Tris_Cl, pH 7.5/120 mM NaCl/25 mM KCl/2 mM EGTA/0.1 mM DTT/0.5% Triton X-100/10 mg/ml leupeptin/0.5 mM PMSF. Total protein (50 µg) from each sample was separated on a 4-20% Tris-HCl Criterion precast gel Bio-Rad (cat# 345-0032, Hercules, CA) and transferred to a poly (vinylidene difluoride) filter (Millipore). The filter was blocked in 5% nonfat dry milk, incubated with the specific antibody, washed, and probed with secondary antibody IgG conjugated to horseradish peroxidase (Santa Cruz Biotechnology), and developed with enhanced chemiluminescence (Amersham Pharmacia). Immunoblot analyses were performed using the following antibodies: Lin28B (Cat#4196, Cell Signaling), Lin28A (Cat#3695, Cell Signaling), Fhit (Cat#71-9000, ThermoFisher Scientific), and Vinculin (Cat# sc-73614, SantaCruz).

### Nanostring array

The NanoString nCounter Human miRNA expression Assay Kit (http://www.nanostring.com) was used to profile more than 700 human and human-viral miRNAs in U87 cells treated with Nutlin-3a (10 µM) and DMSO. 100 ng of total RNA was used as input for nCounter miRNA sample preparation reactions. All sample preparation was performed according to manufacturer's instructions (NanoString Technologies). Preparation of small RNA samples involves the ligation of a specific DNA tag onto the 3′ end of each mature miRNA. These tags are designed to normalize the Tm's of the miRNAs as well as to provide a unique identification for each miRNA species in the samples. The tagging is accomplished in a multiplexed ligation reaction using reverse-complementary bridge oligonucleotides to direct the ligation of each miRNA to its designated tag. Following the ligation reaction, excess tags and bridges are removed, and the resulting material is hybridized with a panel of miRNA: tag-specific nCounter capture and barcoded reporter probes. Hybridization reactions were performed according to manufacturer's instructions with 5 µl of the 5-fold diluted sample preparation reaction. All hybridization reactions were incubated at 64°C for a minimum of 18 hr. Hybridized probes were purified using the nCounter Prep Station (NanoString Technologies) following the manufacturer's instructions to remove excess capture and reporter probes and to immobilize transcript-specific ternary complexes on a sterptavidin-coated cartridge. Date collection was carried out on the nCounter Digital Analyzer (NanoString Technologies) following the manufacturer's instructions to count individual fluorescent barcodes and quantify target RNA molecules present in each sample. For each assay, a high density (600 fields of view) was performed.

### RNA extraction and RT-PCR

Total RNA was extracted using TRIzol Reagent Invitrogen (Cat# 15596-018) following the manufacture's instruction. Specifically, the pellet obtained from 5×10^6^ cells was lysed 1 ml of TRIzol solution. At the end of the extraction the isolated RNA was dissolved in 35 µl in RNase-free water and incubated for 10 min at 55°C. An aliquot of 5 µg RNA was then used for cDNA synthesis using the SuperScript first strand cDNA synthesis kit (Invitrogen). RT-PCRs were carried out using ABI Prism 7900HT sequence detection systems with Applied Biosystems TaqMan Gene expression assays (miR-20a: 4426961; miR-20b: 44426961; let-7a: 4426961; let-7d: 4351372; let-7g: 4426961; FHIT: Hs00896863_m1; LIN28A: Hs00702808_s1; LIN28B: Hs01013729_m1; VDR: Hs01045843_m1; c-JUN: Hs01103582_s1; NFYA: Hs00953589_m1; HOXA10: Hs00538183_m1; EST2: Hs01566396_m1; TCF4: Hs00162613_m1; ELF1: Hs01111177_m1; POU2F1: 01552829_m1; STAT1: Hs01013996_m1; GATA2: Hs00231119_m1).

### Statistical analysis

The data shown at current study are representative result with technical replicate. The data was also confirmed by two or more independent biological replicates. The statistical analysis was performed by paired or unpaired student t-test. Only p-value <0.05 was considered significant.

## Results

### Regulation of let-7 miRNAs through Fhit-activated lin28b expression

**Figure 1:**
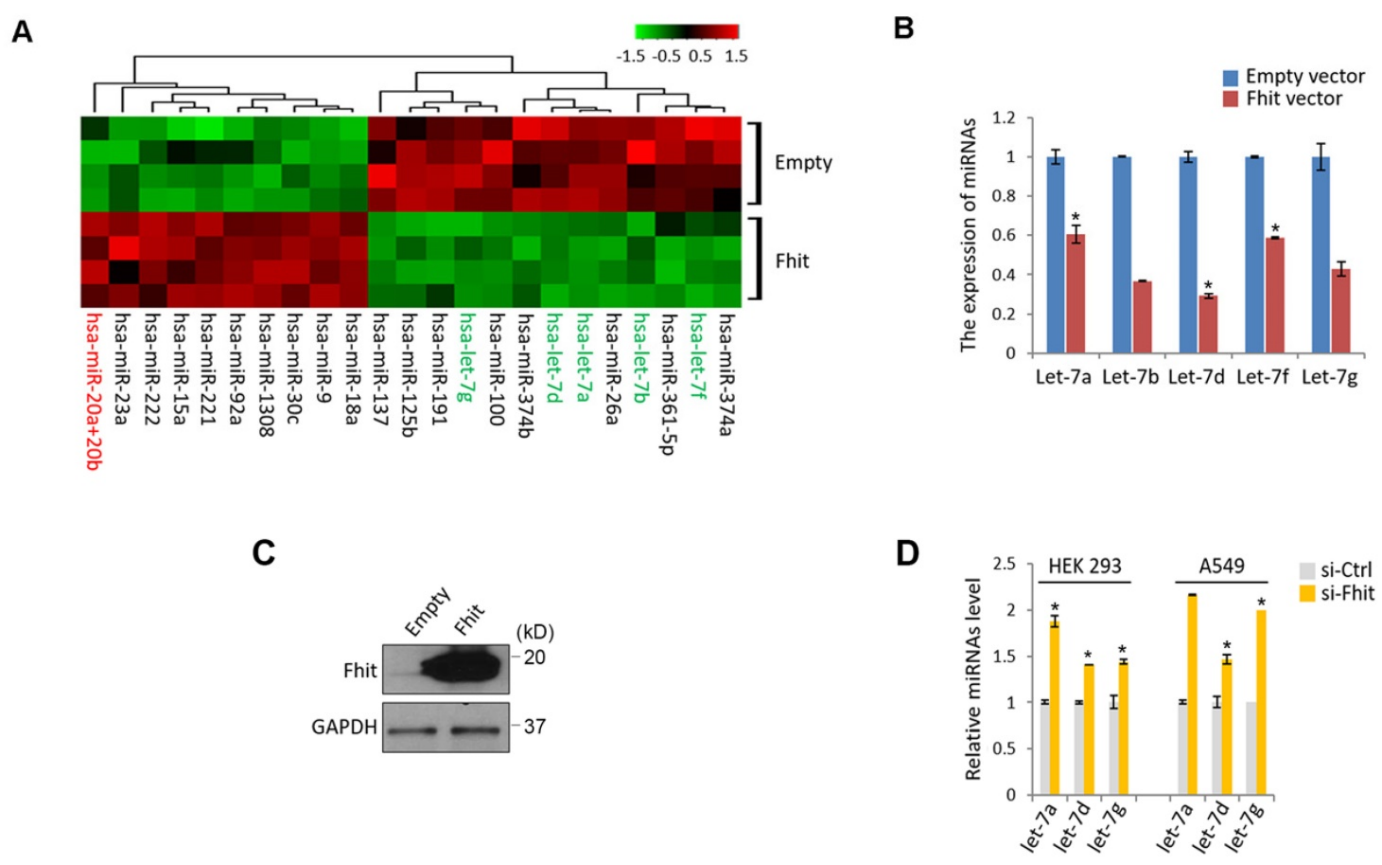
** Suppressed let-7 miRNAs in response to Fhit overexpression.** (A) Unsupervised clustering of miRNAs in HCT116 expressing Fhit. HCT116-Fhit stable cells were generated by transduction of lenti-Fhit virus followed by puromycin selection for 5 days (A). Subsequently, the total RNAs were extracted and analyzed by miRNA microarray analysis. b and c, Validation of let-7 miRNA suppression by Fhit gene in HCT116 cell line. The cells were transiently transfected by Fhit plasmid for 24 hrs. Subsequently the cells were harvested and prepared for qRT-PCR analysis. (B). In parallel the Fhit expression was validated by Western blot analysis using anti-Fhit antibody (C). (D) The suppression of let-7 family by Fhit deficiency in A549 lung cancer cell line enforced by siFhit siRNA treatment. Three independent experiments were performed in triplicate (n=3). Bars mean ±S.D. and the p-value were obtained by student t-test (*p<0.05).

To get a mechanistic insight for Fhit-induced signaling pathway, we genetically engineered to generate a colorectal cancer cell, HCT116 overexpressing Fhit gene, followed by subject the samples to the miRNA nanostring array. An unsupervised clustering analysis shown by heatmap suggested that a bunch of differentially expressed miRNAs were clearly separated and isolated (Supplementary Table 1; Figure 1A). Of those miRNAs, we were interested in miR-17-92 cluster and let-7 family because the miRNAs were redundantly isolated from the same miRNA family (miR-20ab and miR-92a as a miR-17-92 cluster, let-7a,b,d,f and g as a let-7 family), ruling out the feasibility of the false positive. The suppressed expression of let-7 family was confirmed by a stable overexpression of Fhit gene in the HCT116 cells (Figures 1B and 1C). Conversely, Fhit deficient A549 cell lines by siFhit siRNA treatment showed an increase in the expression of the let-7 family, further confirmed in an immortalized embryonic fibroblast, HEK293 cells (Figure 1D).

**Figure 2:**
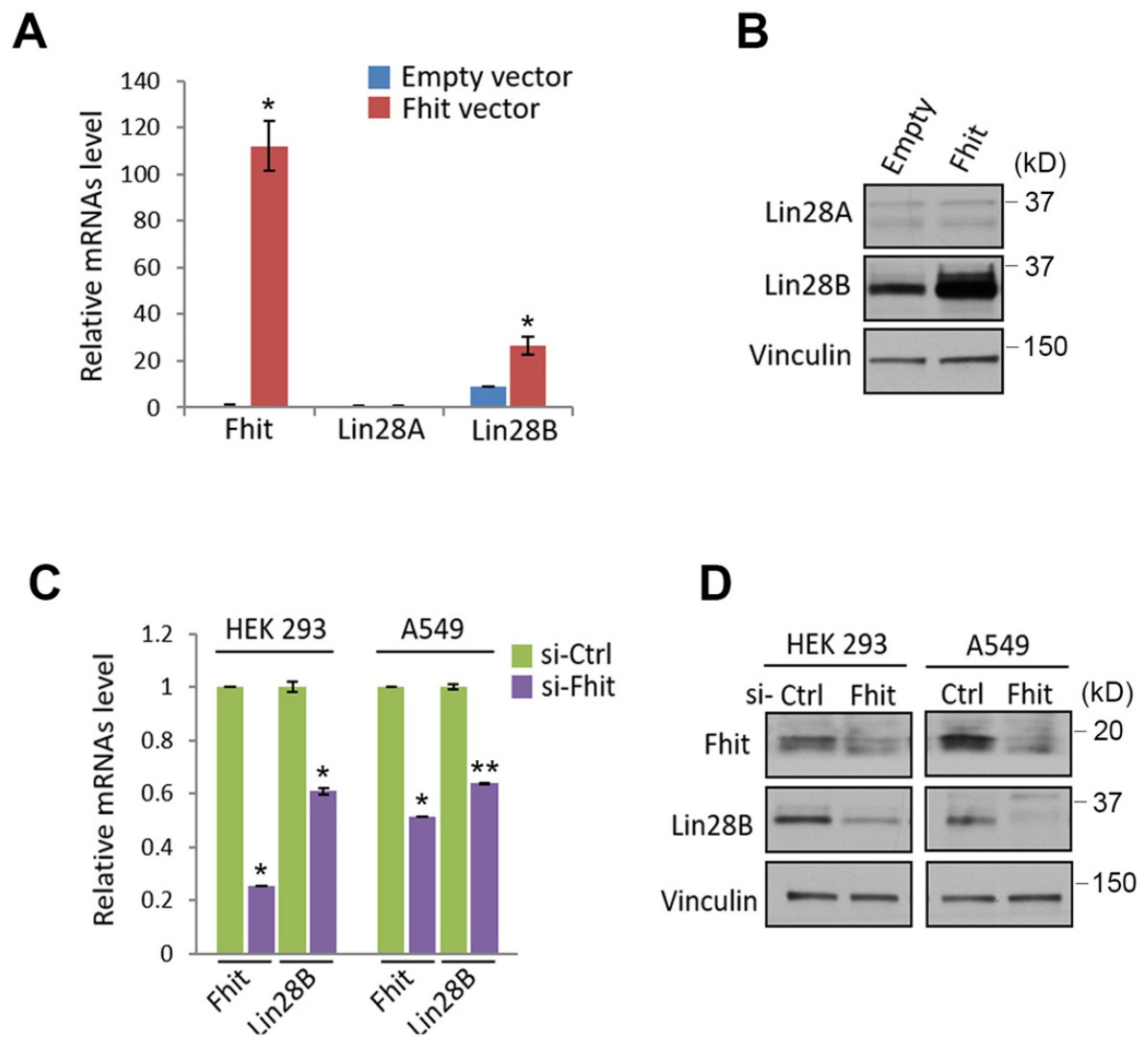
** Fhit-dependent induction of Lin28 protein.** (A) Increased Lin28B mRNA in A549 expressing Fhit gene. The cell was transiently transfected by pcDNA3-Fhit plasmid, and Lin28 mRNA expression was analyzed by qRT-PCR analysis using the extracted RNA from A549 expressing Fhit. (B) Increased Lin28B protein in HCT116-Fhit stable cell line. The indicated cells and its parental cell were prepared and subject to Western blot analysis using anti-Lin28B antibody. Lin28B expression was further validated by independent biological replicate. (C and D) Negative regulation of Lin28 in Fhit deficient cell lines. The indicated cells were transiently transfected by siFhit siRNAs. After 48 hrs of transfection, the cells were harvested and Lin28 expression was analyzed by qRT-PCR (C) or Western blot analysis (D). Three independent experiments were performed in triplicate (n=3). Bars mean ±S.D. and the p-value were obtained by student t-test (*p<0.05; **p<0.01).

Given previous observation that Lin28A/B is one of the best characterized suppressors for let-7 miRNA family, we sought to analyze whether lin28 protein could be regulated by Fhit protein. Indeed, the overexpression of fhit gene in H1299 lung cancer cell enhanced Lin28b expression at both mRNA and protein levels (Figures 2A and 2B). Conversely, the Fhit deficiency enforced by siFhit siRNAs treatment suppressed Lin28 mRNA expression, further confirmed by Western blot analysis showing consistent suppression of Lin28b protein (Figures 2C and 2D).

### Analysis of transcriptional factors to regulate Lin28 in Fhit-overexpressing cells

**Figure 3:**
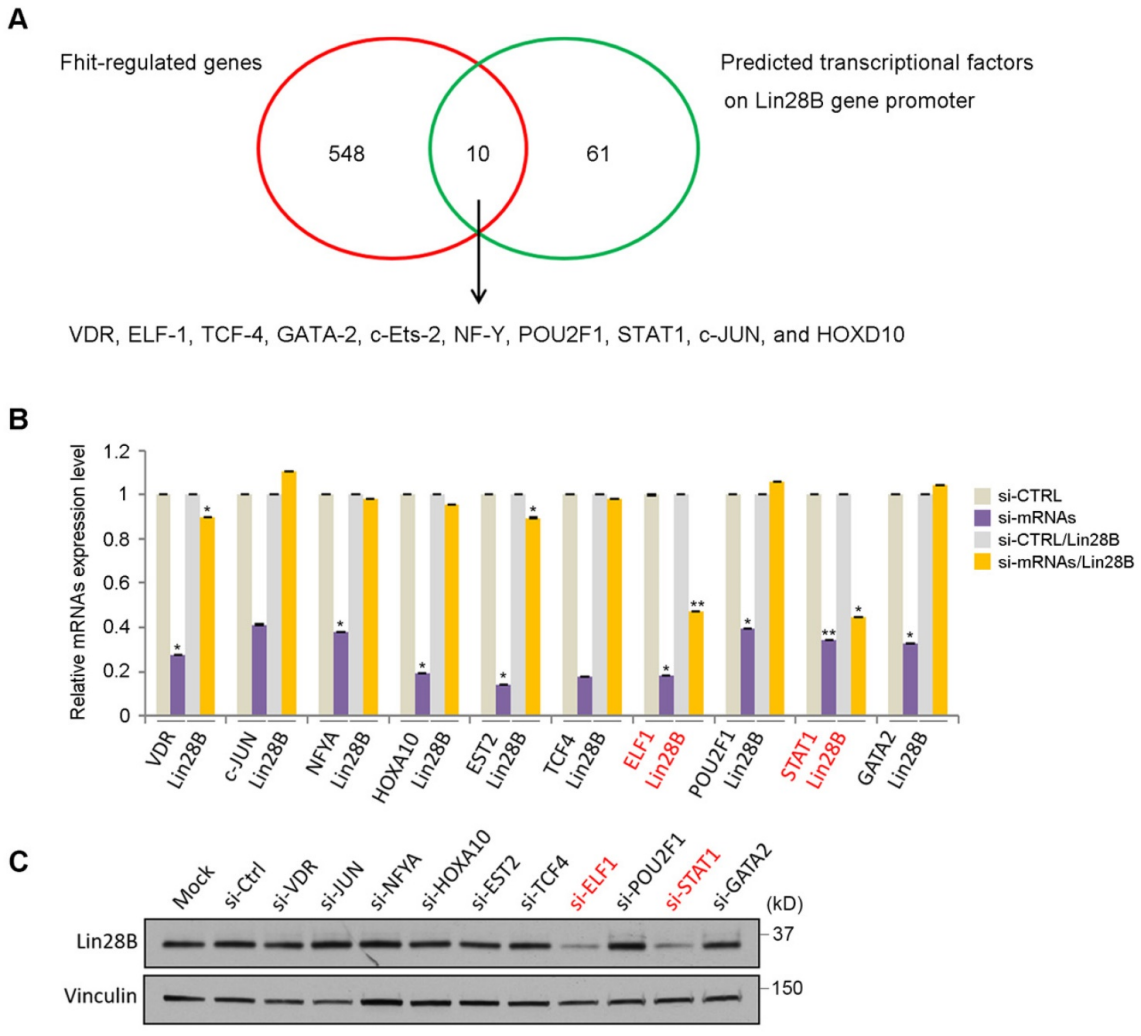
** Identification of transcription factors responsible for Lin28 induction by Fhit.** (A) Schematic diagram showing how the transcription factors for Lin28 induction were identified. Initial 558 candidates were initially isolated by DNA microarray from HCT116-Fhit stablecells compared to its parental HCT 116 cells (red circle). Additional 71 candidates were selected as a predicted transcription factor binding to Lin28 promoter using the ConTra v3 web server which is freely available at http://bioit2.irc.ugent.be/contra/v3. Finally, 10 putative positive transcription factors were identified for subsequent analysis. (B-C) Validation of Lin28b expression in knockdown of each transcription factors by qRT-PCR (B) and Western blot (C). Three independent experiments were performed in triplicate (n=3). Bars mean ±S.D. and the p-value were obtained by student t-test (*p<0.05; **p<0.01).

To analyze how Fhit gene regulates Lin28 expression, we performed DNA microarray analysis using HCT116 expressing Fhit gene and found 558 genes were potentially regulated by Fhit gene (Supplementary Table 2). In addition to that, we also predicted the transcription factors possibly binding to the Lin28b promoter regions. Finally, we identified 10 transcription factors putatively mediating Fhit-dependent Lin28b expression by analyzing the genes commonly observed in both criteria such as Fhit-regulated genes and the transcription factors binding Lin28b promoter (Figure 3A). To validate the profile data, we performed quantitative real-time PCR analysis for 10 putative transcription factors, using RNAs from Fhit-expressing HCT116. We confirmed that their expression levels were higher in Fhit-expressing cells *vs* control cells (Supplementary Figure 1). The qRT-PCR analysis for the 10 putative target transcription factors showed that the Lin28 mRNA was decreased in response to the suppression of the ELF1 or STAT1 expression mediated by their corresponding siRNA treatment, confirmed by Western blot analysis for the protein expression (Figures 3B and 3C). Also, putative binding sites for both transcription factors promoter were found in Lin28b promoter (Supplementary Figure 2). These results suggest that the ELF1 and/or STAT1 are responsible for Lin28b expression in response to Fhit gene overexpression.

### Regulation of miR-17-92 cluster by Fhit gene

**Figure 4:**
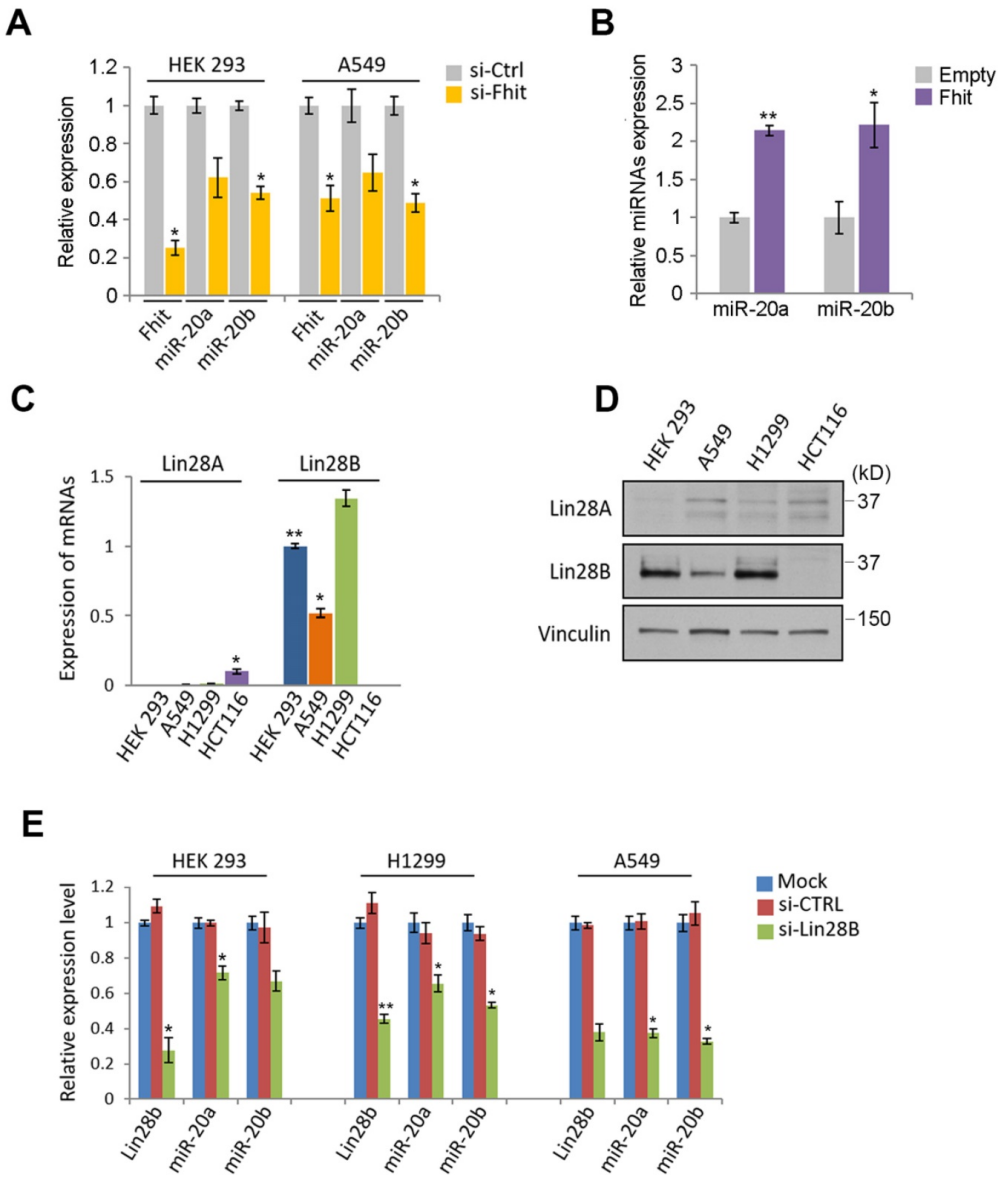
** The suppression of miR-17/92 cluster by Fhit-Lin28 axis. (A and B) The regulation of miR-20a and -20b by Fhit gene**. qRT-PCR analysis showing the suppressed expression of miR-20a and -20b miRNAs in Fhit-deficient HEK293 and A549 cells (A) whereas enhanced expression in A549 expressing Fhit cDNA. (C and D) Analysis of cell lines expressing Lin28. The indicated cells were harvested and prepared for qRT-PCR (C) or Western blot analysis (D). The results were confirmed in an independent biological replicated experiment. (E) Suppressed miR-20a and -20b in response to Lin28b deficiency. The indicated cell lines were transiently transfected by siLin28 siRNAs for 48 hours. Subsequently total RNA was extracted and subject to qRT-PCR analysis. The knock-down efficiency was also shown at left panel of each group of data. Three independent experiments were performed in triplicate (n=3). Bars mean ±S.D. and the p-value were obtained by student t-test (*p<0.05; **p<0.01).

**Figure 5:**
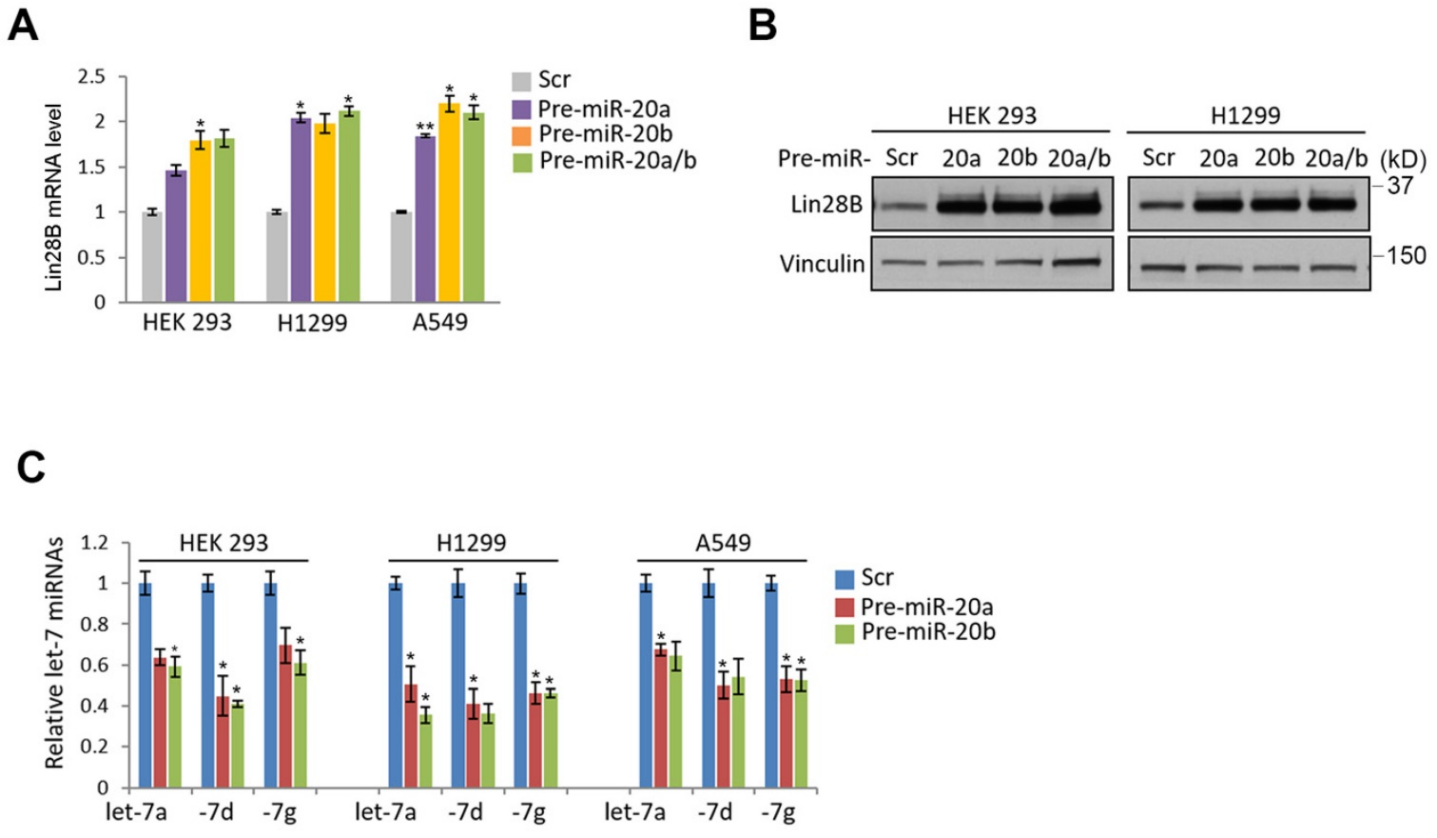
** miR-20a and -20b induction responsible for regulating Lin28B-Let7 axis.** (A and B) Enhanced expression of Lin28B in the cells expressing miR-20a and -20b. The indicated cells were transiently overexpressed by precursors of miR-20a and/or miR-20b. After 48 hours, the expression of Lin28B was analyzed by qRT-PCR (A) or Western blot (B) analysis. (C) Suppressed let7 expression by the ectopic expression of miR-20a and -20b. The cells were transiently transfected by miR-20a and -20b as indicated. After 48 hours, the cells were prepared and subject to qRT-PCR analysis. Three independent experiments were performed in triplicate (n=3). Bars mean ±S.D. and the p-value were obtained by student t-test (*p<0.05; **p<0.01).

Given the nanostring analysis for miRNAs using Fhit-overexpressing HCT116, we also recognized the expression of miR-17-92 clusters including miR-20a, -20b and -92 were enhanced in fhit-overexpressing HCT116 cells (Figure 1A). To analyze whether the regulation of the miR-17-92 clusters by Fhit gene overexpression, we performed the qRT-PCR analysis after knock-down the Fhit gene using siFhit siRNAs, and found miR-20a and miR-20b expression was suppressed by Fhit gene suppression in both HEK293 and A549 cells (Figure 4A). The ectopic expression of Fhit gene in A549 cells conversely showed an increased expression of miR-20a and miR-20b (Figure 4B). To further confirm the Fhit-dependent miR-20a/b regulation, we analyzed the endogenous expression of both Lin28A and Lin28B expression, and found that Lin28B is abundant in A549 and H1299 lung cancer cells and HEK293 cell compared to HCT116 colorectal cancer cell. Unlike Lin28B, no cell line that we tested significantly expressed (Figure 4C and 4D). Given the endogenous expression analysis of Lin28B, we selected the cell lines having abundant lin28B expression, and analyzed miR-20a and miR-20b expression in the context of the Lin28B deficiency. The qRT-PCR analysis showed that both miR-20a and miR-20b expression was suppressed in Lin28B deficient cells (Figure 4E). These data suggest that Fhit enhances miR-20a and miR-20b expression through Lin28B. To our surprise, the overexpression of miR-20a and miR-20b enhanced the mRNA expression of lin28B, further confirmed by Western blot analysis showing consistent induction of Lin28B in either or both miR-20a and/or miR-20b overexpression (Figure 5B and 5C). Taken together, Fhit regulates both miR-17-92 cluster and let-7 family through the modulation of Lin28b expression, and there is a reciprocal regulation between miR17-92 cluster and let-7 family expression.

## Discussion

Since identification of Fhit gene as a LOH gene in chromosome 3p14.2, there were arguments whether Fhit gene was a typical tumor suppressor or just a bystander gene with the chromosome 3p14.2. One of the main challenging points to define Fhit gene as a tumor suppressor was no obvious mechanistic evidence of its physiological function as a nucleotide phosphatase with cancer progression as well as lack of information about either oncogenic or tumor suppressive protein physically interacted with Fhit protein. Moreover, very rare case harboring mutations on coding sequence of Fhit transcripts found in cancer and cancer cell lines 3, 19. However, several lines of evidence have suggested that Fhit transcripts are often dysregulated in multiple cancer as well as cancer cell lines. Moreover, aberrant methylation on promoter of Fhit gene was frequently observed in NSCLC patient and breast primary cancer, supporting the hypothesis that Fhit mRNA expression represent LOH feature 1, 2, 20, 21.

To identify a novel Fhit-induced biology, we employed HCT116 overexpressing Fhit gene and performed microRNA microarray. To our surprise, we identified Fhit induced well established tumor suppressive miRNA, Let-7 family. Moreover, miR-20a, -20b and -92a better known as miR-17/92 clusters were also identified as an induced miRNA by Fhit overexpression. Fhit Lin28/ Let-7 miRNA axis has been known to enhance cancer progression by inhibiting several oncogenic driver gene in different cancer 12, 13. Additionally, miR-17/92 containing 6 different miRNAs are also often found to be increased in several cancer tissues, which consequently plays an important role in cancer progression by targeting several tumor suppressive pathways 15, 16.

Regarding previous literatures, the initial results are very interesting to us because directionality driven by an established tumor suppressor, Fhit gene was opposite such as the suppression of Let-7 family and the induction of miR-17/92 cluster (Figure 1). To further confirm this interesting observation, we characterized that Lin28b known as a suppressor for Let-7 miRNAs is regulated by Fhit gene in lung cancer or colorectal cancer cell line (Figure 2). Additionally, we performed another microarray to identify transcription factors responsible for Lin28B induction, and compared its putative candidates with in silico data. As a result, we found ELF1 and STAT1 transcription factors are responsible for Lin28B protein expression, consistent with previous observations 22, 23, suggesting that Fhit-dependent Let-7 miRNA suppression could be mediated by the induction of Lin28B in response to Fhit overexpression. Moreover, we also characterized there is an inverse correlation between miR-17/92 clusters and Lin28/Let-7 axis driven by Fhit overexpression. Interestingly, Fhit is responsible for the induction of miR-20a and -20b, but the suppression of Let-7 family mediated by Lin28b protein in the same context, which could be mutually exclusive in lung cancer although mechanistic validation should be further analyzed (Figure 4 and 5) 18.

At this point, we do not understand what the physiological relevance of Fhit-dependent Let-7 miRNA suppression is. However, it could be ruled out the feasibility the oncogenic role of Fhit-induced biology. In previous papers, we already confirmed the robust tumor suppressive role by Fhit gene in our experimental system. The overexpression of Fhit induced miR-30c targeting several metastatic and epithelial maker proteins, which consequently enhanced EMT phenotype as well as metastatic potential. Additionally, the anti-metastatic and -oxidative stresses roles by Fhit gene in advanced stage of cancer has been multiply validated *in vitro* and *in vivo* 5, 25. Therefore, we do not believe our current perplexing observation represent an oncogenic role of Fhit gene. In fact, it is generally acceptable notion that a protein has a dual function in a different spatial and/or time context in cancer progression. A proto-oncogene, Myc is a good example for such the paradoxical dual role. Myc is frequently addicted in several solid tumors with high mutation rates to induce genomic instability by regulating gamma-H2AX-dependent DNA damage response. However, the oncogenic Myc can also induce chemo-resistance against DNA-damaging agent 25, 26. Therefore, we believe Fhit-dependent oncogenic regulation should be further investigated to get a better insight for Fhit-induced tumor suppression. Here we show an interesting unrecognized role of Fhit gene to suppress Lin28B/Let-7 axis and enhance miR-17/92 expression, which could be another aspect of Fhit-dependent biology and its physiological relevance has to be recognized to provide mechanistic insight for Fhit gene for anti-cancer therapeutics.

## Conclusions

To elucidate mechanism underlying Fhit-induced tumor suppressive effect, we employed miRNA nanostring analysis, and found the differential expression of let-7 and miR-17/92 clusters. A series of in vitro experiment suggests that ELF-1- and/ or STAT1-dependent Lin28b regulation is responsible for Let-7 induction in Fhit-expressing cancer cells. However, Fhit suppresses the miRNAs of miR-17/-92 clusters and Let-7 in Fhit-expressing cancers, which should be further investigated with a clinical insight.

## Supplementary Material

Supplementary figures.

Supplementary table 1.

Supplementary table 2.
