# Supplementary material for: Fhit induces the reciprocal suppressions between Lin28/Let-7 and miR-17/92miR
Source: Int J Med Sci. 2021 Jan 1;18(3):706–14. doi: 10.7150/ijms.51429 (PMC7797533; doi:10.7150/ijms.51429)
Supplement: Supplementary file 1 — Supplementary figures. [file ijmsv18p0706s1.pdf]

Supplementary Information

Supplementary Figure 1. qRT-PCR analysis showing the expression of 10 transcription factors putatively mediating Fhit-dependent Lin 28b in Fhit-overexpressing HCT116 cells. Three independent experiments were performed in triplicate (n=3). Bars mean ±S.D. and the p-value were obtained by student t-test (*$p<0.05$; **$p<0.01$).

Supplementary Figure 2. Putative transcriptional factor binding sites in Lin28 promoter

Supplementary Table 1. Profiling of microRNAs in Fhit-overexpressing cells

Supplementary Table 2. Profiling of Fhit-regulated transcriptional factors

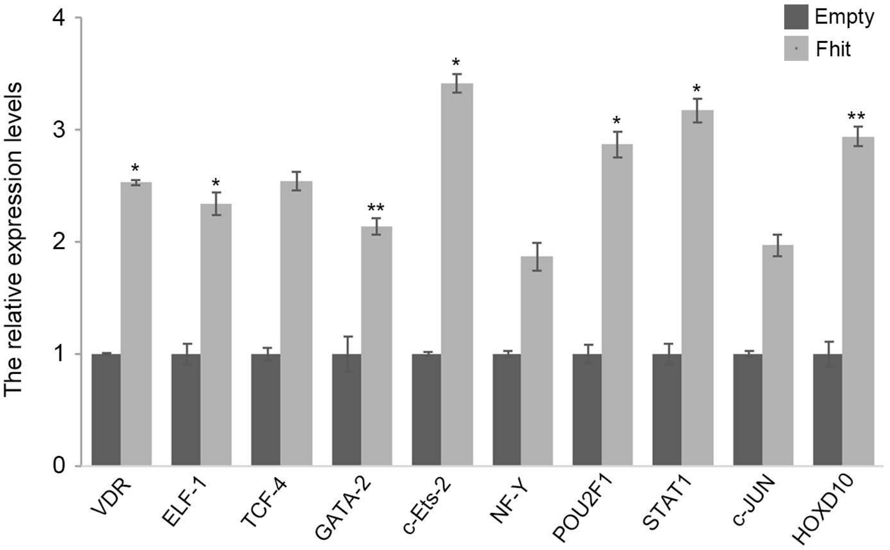

4
3
2
1
0
The relative expression levels
Empty
Fhit
*
*
**
*
*
*
**
VDR
ELF-1
TCF-4
GATA-2
c-Ets-2
NF-Y
POU2F1
STAT1
c-JUN
HOXD10

Consensus sequence and matrix:

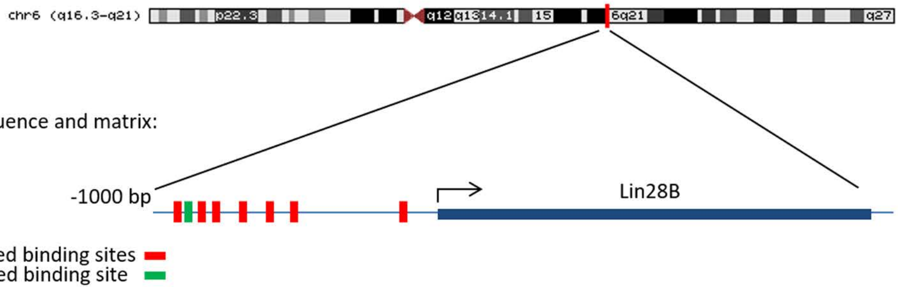

STAT1 predicted sites

Sequence	GGCTGGAAAA		CCCCGGAAAG		TTTTCCAAAA		TTTTCCTAGG		TTTTCCTCTA		AAGGGGAAAA		AGAAGGAAAG	
position	145	154	194	203	255	264	380	389	461	470	537	546	947	956

ELF1 predicted site

Sequence	ATTTAGGAAGTCA	
position	165	177
